# A localized outbreak of Chikungunya virus in Salvador, Bahia, Brazil

**DOI:** 10.1590/0074-02760180597

**Published:** 2019-02-28

**Authors:** Laura B Tauro, Cristiane W Cardoso, Raquel L Souza, Leile CJ Nascimento, Daniela R dos Santos, Gubio S Campos, Silvia Sardi, Olivete B dos Reis, Mitermayer G Reis, Uriel Kitron, Guilherme S Ribeiro

**Affiliations:** 1 Fundação Oswaldo Cruz-Fiocruz, Instituto Gonçalo Moniz, Salvador, BA, Brasil; 2 Instituto de Biologia Subtropical, CONICET, Puerto Iguazú, Misiones, Argentina; 3 Secretaria Municipal da Saúde de Salvador, Salvador, BA, Brasil; 4 Universidade Federal da Bahia, Instituto de Ciências da Saúde, Salvador, BA, Brasil; 5 Yale University, New Haven, CT, USA; 6 Universidade Federal da Bahia, Faculdade de Medicina, Salvador, BA, Brasil; 7 Emory University, Atlanta, GA, USA

**Keywords:** Chikungunya virus, Alphavirus, Outbreak

## Abstract

A localized Chikungunya virus (CHIKV; East/Central/South African genotype) outbreak (50 cases, 70% laboratory-confirmed; attack rate: 5.3 confirmed cases/100 people) occurred in a Salvador, Brazil neighborhood, between Apr-Jun/2017. Highly clustered cases in space and time, mostly along a single street, highlight an increased risk of CHIKV transmission among pockets of susceptible populations. This finding underscores the need for ongoing local level surveillance for arboviral outbreaks.

Chikungunya virus (CHIKV), an alphavirus transmitted by *Aedes* mosquitoes, has become a serious public health problem in Brazil. It was first detected in the country in September 2014 and spread rapidly, joining the long-established Dengue virus (DENV) and the nearly concomitantly introduced Zika virus (ZIKV).^1^ ^,^ ^2^ Salvador, the fourth largest city of Brazil, where DENV has been transmitted endemically since 1995, experienced concomitant outbreaks of CHIKV and ZIKV in 2015.^2^ ^,^ ^3^ ^,^ ^4^ However, since 2016, CHIKV transmission in Salvador has been low, with 657 cases reported (less than half of the 1,332 cases reported in 2015).^5^ Herein, we describe the investigation of a localized CHIKV outbreak that occurred in suburban Salvador in 2017.

On 18 May 2017, a case of fever, arthralgia and other symptoms compatible with an arboviral infection was reported to the Epidemiologic Surveillance Office of Salvador.^3^ Four days later, investigations were initiated where the reported case resided and, by 25 May 2017, 39 cases with similar symptoms have been reported. All of them lived in a small area within Coutos, a poor suburban neighborhood of Salvador (Fig. 1), characterized by a disorganized spatial distribution of self-built houses, which do not have screens on doors or windows, neither have regular water supply or closed sewage pipes. The area is adjacent to the sea and borders the train tracks, where garbage and various mosquito-producing containers were abundant.

Between June and July 2017, we visited all 230 households in the area to detect additional cases presenting fever and arthralgia during the previous 30 days, and among the 662 residents that were counted, 11 additional cases were detected, totaling 50 arboviral suspected cases, living in 33 households. Clinical data were collected from all the 50 cases, and blood samples from 45 (90%) of them.

**Fig. 1:**
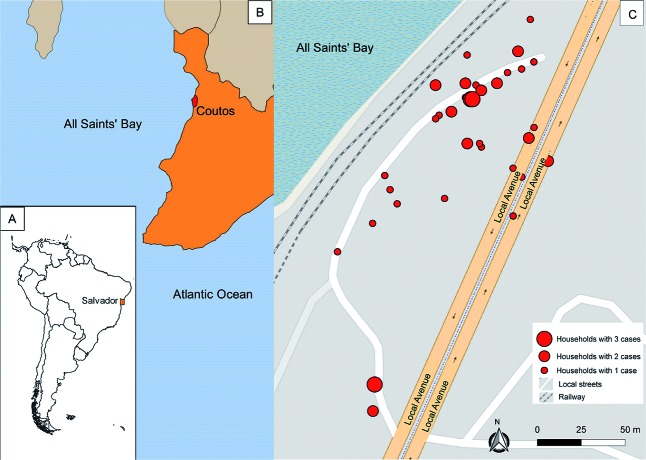
spatial distribution of households of Chikungunya cases during an outbreak in Coutos neighborhood, Salvador, Bahia (BA), Brazil. (A) Location of Salvador in Brazil. (B) Location of Coutos neighborhood in Salvador. (C) Spatial distribution of the households of chikungunya cases (mostly along a side street situated between a railway line and a larger avenue).

As the initial clinical suspicion was CHIKV or DENV infection, serum samples were first tested by IgM enzyme-linked immunosorbent assay (ELISA) for CHIKV (Euroimmun, Germany) and DENV (Focus Diagnostics, USA). In addition, RNA was extracted from 17 cases with an available sample stored at -70ºC and amplified by reverse transcription polymerase chain reaction (RT-PCR) using primers for CHIKV, ZIKV, and DENV, as well as for Oropouche (OROV), Mayaro (MAYV), and Yellow Fever virus (YFV).^6^ ^,^ ^7^ ^,^ ^8^ ^,^ ^9^ ^,^ ^10^ ^,^ ^11^ The PCR products were sequenced using the Sanger method.

To investigate mosquitoes species potentially involved in the outbreak, we surveyed the household of suspected cases. Pools of captured female mosquitoes from each species were tested by RT-PCR for the same arboviruses as described above. Virus isolation was also attempted in C6/36 (*Aedes albopictus*) cell cultures.

Of the 45 patients tested by CHIKV IgM ELISA, 35 (77.8%) were positive, eight (17.8%) negative, and two (4.4%) equivocal. IgM ELISA testing for DENV was performed for 31 (68.9%) of the 45 samples and three (9.7%) were positive, 25 (80.6%) negative, and three (9.7%) equivocal. Of the three patients IgM-positive for DENV, two were also positive for CHIKV. Of the 17 patients tested by RT-PCR, eight (47.1%) were positive for CHIKV. All of them were also positive by the CHIKV IgM ELISA. No other arbovirus was detected by RT-PCR.

We sequenced the PCR products of seven CHIKV confirmed cases and the consensus sequences (MG591452, and MG591454 to MG591458) showed a nucleotide identity of 100% among them, and 99% with the sequences previously obtained in the state of Bahia, in the cities of Feira de Santana in 2014 (KP164568-KP164570) and Salvador (KU940225) in 2015, all belonging to the East/Central/South African (ECSA) genotype.^1^ ^,^ ^12^ Phylogenetic analysis was made using Mega version 7.0 software by means of the Neighbour joining method and a p-distance model bootstrapped 1,000 times (Fig. 2).

**Fig. 2:**
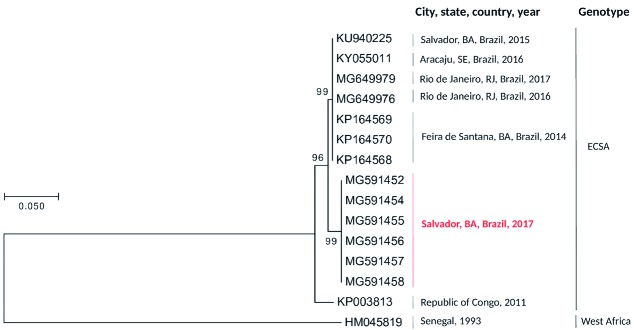
consensus tree generated from nucleotide sequences of a fragment of 300 bp of the E2 protein gen, using the Neighbour joining method and a p-distance model bootstrapped 1,000 times. The sequences (MG591452, and MG591454 to MG591458) obtained in this study are highlighted in red. A West Africa Chikungunya virus (CHIKV) sequence (HM045819) is included as an outgroup. ECSA: East/Central/South African; BA: Bahia; SE: Sergipe; RJ: Rio de Janeiro.

The 50 cases had a median age of 38 years, and 23 (46%) were females (Table). Clinical characteristics of the laboratory-confirmed and unconfirmed cases were similar, except for rash, which were 2.5-fold times more common among the confirmed group, but this difference was not statistically significant (p = 0.06) (Table). Arthralgia was present in all the 50 cases and 45 (95%) reported it to be symmetric and polyarticular. None of the patients was hospitalized.

The overall attack rate was 7.6 cases/100 persons (8.5 cases/100 men; 6.4 cases/100 women; 5.4 cases/100 children < 15 years of age; 7.3 cases/100 persons 15-39 years of age, and 9.0 cases/100 adults ≥ 40 years of age). Considering only the laboratory-confirmed CHIKV cases, the attack rate was 5.3 cases/100 persons (5.7 cases/100 men; 4.9 cases/100 women; 0.7 cases/100 children < 15 years of age; 5.4 cases/100 persons 15-39 years of age, and 5.6 cases/100 adults ≥ 40 years of age). Of the 50 cases, 45 (90%) resided in the same street (Fig. 1).

**TABLE:** and clinical characteristics of patients suspected of Chikugunya virus (CHIKV) infection during a community outbreak in Salvador, Brazil, according to CHIKV laboratory test results, April to June 2017

Reported characteristics	Total suspected cases (n = 50)	Laboratory-confirmed cases^*a*^ (n = 35)	Unconfirmed cases^*b*^ (n = 15)	p-value^*c*^
	Number (%) or median (interquartile range)				
Demographic				
Female	23 (46)	17 (48)	6 (40)	0.75
Median age	38 (23 - 48)	38 (23 - 48)	42 (28 - 48)	0.84
Clinical				
Fever	50 (100)	35 (100)	15 (100)	1.00
Arthralgia	50 (100)	35 (100)	15 (100)	1.00
Polyarticular^*d*^	45 (90)	32 (91)	13 (86)	0.62
Symmetric^*e*^	45 (90)	31 (88)	14 (93)	1.00
Myalgia	49 (98)	35 (100)	14 (93)	0.30
Prostration	43 (86)	31 (88)	12 (80)	0.41
Chills^*f*^	39 (79)	25 (74)	14 (93)	0.14
Headache	36 (72)	26 (76)	10 (66)	0.50
Retro-orbital pain	30 (60)	20 (57)	10 (66)	0.75
Pruritus^*g*^	27 (56)	18 (54)	9 (60)	0.76
Joint edema	24 (48)	17 (48)	7 (46)	1.00
Nausea	22 (44)	15 (42)	7 (46)	1.00
Rash	21 (42)	18 (51)	3 (20)	0.06
Conjunctival hyperemia	20 (40)	15 (42)	5 (33)	0.75
Vomit	12 (24)	8 (22)	4 (26)	1.00
Swollen lymph nodes	7 (14)	6 (17)	1 (7)	0.65

*a*: all 35 laboratory-confirmed patients were positive by CHIKV immunoglobulin M (IgM) enzyme-linked immunosorbent assay (ELISA); eight of them were also positive by CHIKV reverse transcription polymerase chain reaction (RT-PCR); *b*: of the 15 unconfirmed patients suspected of CHIKV infection, eight were negative, two equivocal, and five not tested by CHIKV IgM ELISA; *c*: Fisher exact test p-values for the comparisons between confirmed and unconfirmed cases suspected of CHIKV infection; *d*: polyarticular arthralgia defined by pain in more than one joint; *e*: symmetric arthralgia defined by pain in at least one pair of joints; *f*: data not available for one laboratory-confirmed CHIKV infection case; *g*: data not available for two laboratory-confirmed CHIKV infection cases.

The first case initiated symptoms in April, but most cases occurred during May (Fig. 3). The spatial and temporal distribution of all cases was analyzed with a k nearest neighbor (k-NN) statistic for space-time clustering using ClusterSeer software (Biomedware, Ann Arbor, MI).^13^ The k-NN statistic is the number of case pairs that are k^th^ nearest neighbors when both space and time are considered. The null hypothesis is that nearest neighbor relationships in space and time are independent from each other. Cases were highly clustered in space and time, with proximal cases also the ones closest temporally (p < 0.01 for the first nearest neighbors).

**Fig. 3:**
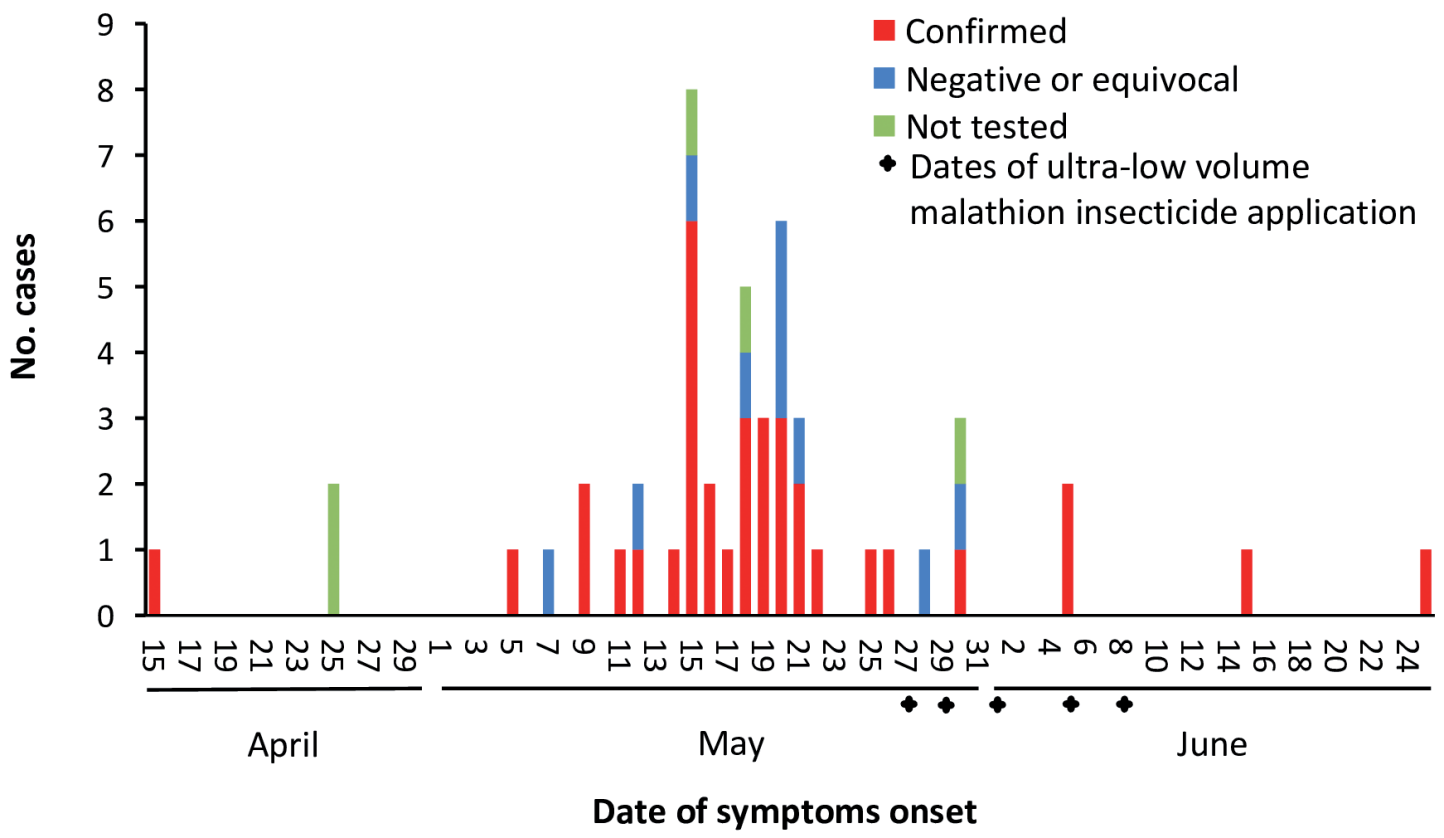
number of cases per day during the Chikungunya virus outbreak in Coutos neighborhood, Salvador, Bahia (BA), Brazil.

We performed entomological surveys in 27 of the 33 houses with suspected cases, between 14 June and 18 July 2017 (Fig. 1). A total of 125 adult mosquitoes were collected in 21 houses, with *Culex quinquefasciatus* the most abundant [99, 79.2%; 69 of them (69.7%) female], followed by *Ae. aegypti* [26, 20.8%; eight of them (30.8%) female]. The RT-PCR and culture isolation from all of them yielded negative results.

After 2.5 years of the first CHIKV detection in Brazil and the ensuing countrywide spread of the virus, we found that CHIKV maintains its potential to cause highly localized outbreaks. It is likely that similar small, circumscribed outbreaks, which are not easily detectable, help explain the prolonged CHIKV transmission in Brazil, in contrast to the more widespread, explosive ZIKV transmission pattern that was observed.^2^

Most of the cases during this outbreak were residents of a single street, and the strong space-time clustering points to an outbreak that travelled from house to neighboring house. This is probably associated with transmission by multiple mosquitoes that became infected at about the same time and/or by a small number of infected mosquitoes feeding on multiple proximal hosts.^14^

Poor socioeconomic conditions in the neighborhood, such as unreliable water supply and waste collection services, and the accumulation of containers that serve as habitats for *Aedes* larvae may have facilitated this outbreak. In response, the Zoonosis Control Center, the municipal agency responsible for mosquito surveillance and control, intervened in the area aiming to reduce mosquito numbers by removing any sources of standing water, treating water-holding containers with larvicides, and outdoor spraying of insecticide in five cycles (Fig. 3). In addition, the community was sensitized to act together to eliminate potential mosquito breeding sites. As many of these interventions were conducted after the outbreak peak, we were not able to determine their role in preventing additional cases. We also could not establish mosquito species implicated in CHIKV transmission during this outbreak because we did not detect infected mosquitoes. This is probably because the number of collected and tested mosquitoes was low due to the insecticide application in the region, which preceded our entomological surveys.

Given the complex epidemiological scenario in the Americas since CHIKV and ZIKV joined DENV as common etiologies of urban febrile diseases, we reinforce the importance of integrating data from clinics, entomological surveys, epidemiological surveillance, and laboratory testing during outbreak investigations and surveillance activities. Only with ongoing local level surveillance of arboviral diseases, outbreaks affecting pockets of susceptible population will be promptly detected, in order to guide timely control measures.
